# Effects of Recreational Football on Body Composition and Cardiometabolic Health in Overweight or Obese Individuals: A Systematic Review and Meta-Analysis

**DOI:** 10.3390/life15081276

**Published:** 2025-08-12

**Authors:** Sijia Li, Haoran Li, Bo Wang, Zhuo Zeng, Rui Zhang, Henghao Yan, Aiguo Zhou, Yongmin Xie, Chengyu Zhou

**Affiliations:** 1 China College of Sports Medicine and Rehabilitation, Beijing Sport University, Beijing 100084, China; flamingo13@foxmail.com; 2 Strength and Conditioning Training College, Beijing Sports University, Beijing 100084, China; marslee_24@163.com (H.L.); 2024210137@bsu.edu.cn (Z.Z.); aiguozhou@126.com (A.Z.); 3 Physical Education College, Jilin University, Changchun 130012, China; wangbo0522@mails.jlu.edu.cn; 4 College of Science, North China University of Technology, Beijing 100144, China; brackzr@ncut.edu.cn; 5 School of Physical Education, Southwest University, Chongqing 400715, China; 15823182337@163.com

**Keywords:** recreational football, obesity, body composition, cardiometabolic health, exercise, meta-analysis

## Abstract

**Objective:** This study systematically examined the effects of recreational football on body composition and cardiometabolic health in overweight or obese individuals via subgroup analyses of potential moderators. **Methods:** A systematic search was conducted across six databases (PubMed, Web of Science, the Cochrane Library, CNKI, VIP, and Wanfang Data) in May and July 2025 to identify controlled trials evaluating recreational football among overweight or obese individuals. A meta-analysis was performed to pool the effect estimates, reported as standardized mean differences (SMDs), with heterogeneity explored through subgroup analyses. **Results:** Recreational football interventions across 32 studies (1126 participants, aged 11–68) led to significant improvements in body composition and cardiometabolic health. The training programs ranged from 4 to 48 weeks, with sessions lasting 4 to 30 min. Key body composition outcomes included reductions in body weight (SMD = −0.51), body mass index (SMD = −0.41), body fat percentage (SMD = −0.53), fat mass (SMD = −0.40), and waist circumference (SMD = −1.43), along with increases in lean body mass (SMD = 0.18). The effects were more pronounced in females and younger participants (≤18 years). Cardiometabolic benefits included reductions in systolic blood pressure (SMD = −0.59), diastolic blood pressure (SMD = −0.75), and mean arterial pressure (SMD = −0.91), as well as resting heart rates (SMD = −0.85), especially among females, obese males, and those subject to shorter rest intervals. Participants’ peak oxygen uptake also improved (SMD = 0.81). Concerning lipid metabolism, participants’ total cholesterol, low-density lipoprotein cholesterol, and triglycerides decreased significantly, particularly in females, younger and obese individuals, and those who trained more than twice per week. High-density lipoprotein cholesterol increased significantly only in females and those involved in frequent training. In regard to glucose metabolism, participants’ fasting insulin declined (SMD = −0.47), especially in regard to programs exceeding 12 weeks, whereas no significant changes were observed in fasting blood glucose, glycated hemoglobin, or the homeostatic model assessment of insulin resistance. According to the GRADE assessments, the certainty of the evidence ranged from very low to moderate across these outcomes. **Conclusions:** Recreational football improves the body composition and cardiometabolic health in overweight or obese individuals, resulting in reductions in adiposity, blood pressure, lipids, and insulin, with greater benefits observed in females, younger individuals, and those engaging in more frequent training. These findings support its potential as a practical intervention for weight and cardiometabolic risk management, in both clinical and community settings.

## 1. Introduction

The global epidemic of overweight and obesity has emerged as one of the most pressing chronic health challenges, affecting over 1.9 billion adults worldwide and placing a substantial burden on public health systems [1,2]. This problem has been further intensified by widespread physical inactivity, unhealthy dietary habits, and increased psychological stress, particularly during and after the COVID-19 pandemic, all of which have contributed to increasing body weight, BMI, and obesity prevalence among adults [3]. A defining feature of overweight and obesity is the ectopic accumulation of fat, especially in no adipose tissues, such as skeletal muscle and the liver [4]. This abnormal fat deposition is closely linked to insulin resistance, impaired skeletal muscle function [5], and a markedly increased risk of cardiometabolic diseases, including type 2 diabetes and cardiovascular disease [1,2]. Although obesity is a multifactorial condition, shaped by genetic, environmental, and behavioral influences, its primary cause remains a chronic imbalance between energy intake and expenditure [6,7].

In this context, structured and sustainable physical activity is widely recognized as a cornerstone strategy for preventing and managing obesity and related metabolic disorders [8]. Among various exercise modalities, recreational football has gained increasing attention because of its unique combination of practicality, high intrinsic motivation, and diverse physical demands [9]. Unlike competitive football, recreational football emphasizes enjoyment and inclusivity, making it accessible to individuals across a wide range of ages, sexes, and fitness levels [9]. It typically involves intermittent bouts of high-intensity activity, such as sprints, directional changes, jumps, and accelerations, that elicit substantial cardiovascular stimulation, muscular recruitment, and energy expenditure. On average, these sessions achieve 80–85% of the maximum heart rate (HR_max_), with significant neuromuscular engagement across multiple joints and major muscle groups [10,11,12].

Although previous research has demonstrated the benefits of recreational football for improving general physical fitness [10,11,12], evidence specifically focused on overweight or obese populations remains limited. Most existing studies have examined isolated outcomes, particularly cardiorespiratory fitness [12], whereas few have conducted comprehensive assessments of body composition and cardiometabolic health indicators, such as glucose and lipid metabolism. Moreover, no prior meta-analysis has systematically investigated subgroup effects in this population to evaluate how participant characteristics (e.g., sex, age) or exercise prescription variables (e.g., frequency, duration) might influence outcomes. This lack of detailed stratification limits the ability to develop more precise, evidence-based exercise recommendations for overweight or obese individuals.

Therefore, this meta-analysis aims to systematically evaluate the effects of recreational football on body composition and cardiometabolic health in overweight or obese individuals and to explore the potential moderating influences of participant characteristics and exercise prescription variables. These findings may inform the development of targeted, scalable exercise interventions for obesity management, in both clinical and community settings.

## 2. Methods

This systematic review adhered to the 2020 Preferred Reporting Items for Systematic Reviews and Meta-Analyses (PRISMA) statement [13]. The completed PRISMA 2020 checklist is available in Appendix A. Additionally; this review has been registered in the OSF database https://osf.io/wvmyj (accessed on 22 June 2025).

### 2.1. Information Sources and Search Strategy

Database searches were conducted in PubMed, Web of Science, the Cochrane Library, as well as three Chinese databases: CNKI (China National Knowledge Infrastructure), the VIP Database for Chinese Technical Periodicals, and Wanfang Data. No restrictions were applied regarding the publication date, participant characteristics, or language, provided that the title and abstract were available in English or Chinese. To ensure comprehensive coverage, three additional search strategies were applied: screening of the reference lists of all the included articles, identifying articles that cited the included studies via citation tracking, and exploring similar articles through the “Similar Articles” feature in PubMed and the “Find Similar” function in Embase. The searches were conducted on 1 May 2025. The search terms were formulated on the basis of the PICOS framework and were categorized into two main concepts: population “overweight”, “obese”; and intervention “recreational football”, “small-sided soccer”, “football training”, “football-based exercise”, and related terms. Additionally, the PROSPERO database and the Cochrane Database of Systematic Reviews were searched to ensure that no similar systematic reviews had already been registered or published. An updated search was conducted on 28 July 2025 to ensure the inclusion of the most recent studies.

### 2.2. Selection Process

Deduplication of the retrieved records was performed manually by independent reviewers via EndNote X9 [Clarivate Analytics, 2018]. The deduplicated records were then exported and provided to two independent researchers, who screened the titles and abstracts of all the articles on the basis of predefined inclusion and exclusion criteria. In cases of disagreement, the two reviewers met to re-examine the eligibility criteria and resolve discrepancies through discussion. If consensus could not be reached, a third independent researcher was consulted to make the final inclusion decision. The same pair of independent reviewers then conducted a full-text review to determine final eligibility. In the event of disagreement at this stage, the same adjudication protocol was applied. Additional potentially relevant sources included reference lists from previous systematic reviews in the field and articles identified through the research team’s domain expertise that may have met the inclusion criteria but were not captured by the initial database search.

### 2.3. Eligibility Criteria

The inclusion and exclusion criteria were developed according to the Population, Intervention, Comparison, Outcome, Study design (PICOS) framework, as detailed below.

The inclusion criteria were population (P): Participants were classified as overweight or obese based on body mass index (BMI) criteria (i.e., BMI ≥ 25 kg/m^2^ for overweight and BMI ≥ 30 kg/m^2^ for obese), regardless of sex, age, baseline physical fitness, or specific health status.

Intervention (I): The intervention consisted of recreational football training, operationally defined as small-sided (typically 4v4), noncompetitive, group-based football sessions, conducted on reduced-size pitches and primarily intended for health promotion and physical fitness. To ensure consistency, studies were included only if the intervention reported a training duration of at least two weeks. Interventions were required to report clear descriptions of the training frequency, intensity, and duration.

Comparison (C): Comparators included non-exercise control, waiting list control, placebo, or usual care, such as nutritional advice or general lifestyle recommendations without structured exercise.

Outcomes (O): Studies were required to report at least one health outcome related to weight management, including, but not limited to, body composition indicators (e.g., body weight, body fat percentage); cardiovascular health measures (e.g., blood pressure, resting heart rate, peak oxygen uptake [VO_2_peak]); and metabolic markers (e.g., blood glucose, insulin sensitivity, lipid profiles).

Study Design (S): Eligible studies were longitudinal controlled intervention trials, including randomized controlled trials (RCTs) and nonrandomized controlled trials (non-RCTs), with quantitative pre- and postintervention assessments, and reported between-group comparisons.

Studies were excluded if they lacked a control group (i.e., single-arm designs), failed to report data separately for overweight or obese participants, or involved interventions unrelated to recreational football, such as other exercise modalities, psychological interventions, or standalone dietary programs. Additional exclusion criteria included nonoriginal research formats (e.g., reviews, methodological articles, conference abstracts, gray literature, case reports, or qualitative studies), studies lacking postintervention outcome data or with insufficient information for effect size extraction, and duplicate publications or datasets, in which case only the version with the most complete and relevant data was retained.

### 2.4. Data Extraction

Data extraction was carried out by the same two reviewers who conducted the screening process, via a customized extraction worksheet developed in Microsoft Excel, prior to the full-text review phase. Both reviewers independently extracted the following information from each included study: author and publication details, study design and characteristics, participant demographics, intervention protocols, and outcome assessments. A third reviewer performed an additional round of cross-checking to verify the accuracy of the data. In cases of disagreement, a fourth independent researcher was consulted to reach a consensus through discussion. If data were missing or presented only in graphical form, the corresponding authors were contacted to request the required information. When author contact was unsuccessful and the data remained available only in figure format, quantitative values were extracted via WebPlotDigitizer version 4.1. If the missing data could not be retrieved through either author contact or graphical extraction, the study was excluded from the final quantitative analysis.

This study extracted the mean, standard deviation, and sample size reported for each group pre- and postintervention. We pooled the effects using pre- and postintervention differences (*M* ± *SD*) for each outcome indicator. The first step was to calculate the difference in the means (raw mean difference between postintervention and preintervention for each intervention group) via the following Formula (1):

(1) $${MD}_{diff\;}=M_{post}\;-M_{pre}\;$$

where ${MD}_{diff}$ represents the raw mean difference, ${MD}_{diff}$ represents the reported mean postintervention, and $M_{pre}$ represents the reported mean preintervention.

If the study reported confidence intervals only, they were converted into SDs via the following Formula (2):

(2) $$SD=\sqrt{N}\frac{{CI}_{high}-{CI}_{low}}{2t}$$

where *SD* is the standard deviation, *N* is the group sample size, ${CI}_{high}$ is the upper limit of the confidence interval, ${CI}_{low}$ is the lower limit of the confidence interval, and *t* is the *t* distribution with *N* − 1 degrees of freedom at the respective confidence level.

The *SD* of the difference in the means (${SD}_{diff}$) was calculated via the following Formula (3) [14]:

(3) $${SD}_{diff}=\sqrt{{{SD}_{pre}}^{2}+{{SD}_{post}}^{2}-2r\times {SD}_{pre}\times {SD}_{post}}$$

where ${SD}_{diff}$ is the standard deviation of the difference in the means, ${SD}_{pre}$ is the standard deviation from the preintervention, and ${SD}_{post}\;$ is the standard deviation from the postintervention. As the original studies included in the meta-analysis did not report Pearson’s correlation coefficients (r) for the pre- and postintervention outcomes, we used 0.5, which was an ${SD}_{change}$, based on the recommendations in the Cochrane Library. It was calculated via Formula (4):

(4) $$r=\frac{{{SD}_{pre}}^{2}+{{SD}_{post}}^{2}-{{SD}_{change}}^{2}}{2\times {SD}_{pre}\times {SD}_{post}}$$

### 2.5. Risk of Bias and Quality of Methods Assessment

The risk of bias was assessed independently by two reviewers via the Cochrane Collaboration’s risk of bias tool 2 (RoB 2). This tool evaluates bias across multiple domains, including random sequence generation, allocation concealment, the blinding of participants and personnel, the blinding of the outcome assessment, incomplete outcome data, selective outcome reporting, and other sources of bias. Disagreements between reviewers were resolved through discussion, whenever possible. If a consensus could not be reached, a third independent reviewer was consulted for adjudication. For nonrandomized studies, the risk of bias was evaluated via the Risk of Bias in Nonrandomized Studies of Interventions (ROBINS-I) tool, which assesses seven domains of bias: confounding, the selection of participants, the classification of interventions, deviations from the intended interventions, missing data, measurement of outcomes, and the selection of the reported result.

Additionally, the Physiotherapy Evidence Database (PEDro) scale was used to assess the methodological quality of the included studies. The PEDro scale rates studies on a scale from 0 to 10, with scores of ≥6 indicating high quality, scores of 4–5 indicating moderate quality, and scores ≤ 3 indicating low quality.

### 2.6. Statistical Analysis

Statistical analyses were conducted via the “meta” and “metafor” packages in R statistical software (version 4.2.1) [14,15]. For the meta-analysis, a generic inverse variance pooling method was employed, and the pooled effect sizes were calculated via a random effects model, using the DerSimonian-Laird approach [16]. Given that the outcome measures in this study often involve various units of measurement, in line with previous research suggestions [17], this study prioritized the use of standardized mean differences (SMDs) as the effect size indicator. Additionally, forest plots summarizing each outcome category were generated via the “orchard” package [18], providing a visual representation of the pooled estimates and heterogeneity across the studies.

Additionally, considering the small sample sizes in most of the included studies, Hedges’ g, which is based on a precise formula, was used as the effect size, and corrections for bias were applied. The Hedges’ g values were categorized as trivial (0.2), small (0.2–0.5), medium (0.5–0.8), or large (>0.8). A variety of metrics, including Cochrane’s Q, the I^2^ statistic, tau^2, and Tau, were employed to evaluate heterogeneity [19]. We computed a 95% confidence interval (CI). The prediction interval (PI) was also calculated to more comprehensively reflect the potential variability in future similar studies, and multiple indicators were reported simultaneously [17]. Recent studies and standard textbooks have predominantly advocated the I^2^ statistic as the principal indicator of heterogeneity. Consequently, our primary analysis utilized I^2^, interpreting its values as follows: 0–25% potentially insignificant; 25–50% indicative of moderate heterogeneity; 50–75% suggestive of substantial heterogeneity; and 75–100% reflective of considerable heterogeneity [20]. To evaluate the statistical power of the pooled effect estimates and minimize type II error, power analyses were conducted via the “metameta” package [21].

Furthermore, subgroup analyses and meta-regressions were conducted to examine potential moderators and explore sources of heterogeneity across both categorical and continuous variables [22]. These analyses focused on three primary domains: participant characteristics, intervention characteristics, and training protocol parameters. On the basis of the theoretical rationale and data availability, six key subgroup variables were selected: (1) sex, (2) age group, (3) weight status, (4) intervention week, (5) training frequency, and (6) rest interval between sets or exercises. To ensure interpretability and sufficient statistical power, subgroup analyses were performed only when each subgroup contained at least five studies [23]. Furthermore, subgroup analysis was considered appropriate only when moderate-to-high heterogeneity (I^2^ > 25%) was observed in regard to the overall pooled effect estimates.

Finally, publication bias was assessed via contour-enhanced funnel plots and Egger’s regression test, with a *p* value > 0.05 considered indicative of no significant publication bias [24]. Sensitivity analysis was performed using a one-study-removed (leave-one-out) method, whereby each study was sequentially excluded to evaluate its influence on the overall pooled estimate.

### 2.7. Certainty of the Evidence

The evidence of effectiveness for each study was combined with the quality scores for use in discussing the results. The Grading of Recommendations Assessment, Development, and Evaluation (GRADE) methodology was used to rate the certainty of the evidence as “high”, “moderate”, “low”, or “very low” [25]. GRADE was completed by two researchers, with differences resolved through consensus. This comprehensive assessment rates evidence as follows: (1) the risk of bias, downgraded by one level if “some concerns” and two levels if “high risk” of bias; (2) inconsistency, downgraded by one level when the impact of statistical heterogeneity (I^2^) is moderate (>25%) and by two levels when high >75%; (3) imprecision, downgraded by one level when statistical power < 80% and if there was no clear direction of the effects [26]; and (4) risk of publication bias, downgrade one level if Egger’s test < 0.05.

## 3. Results

### 3.1. Studies Retrieved

**Figure 1:**
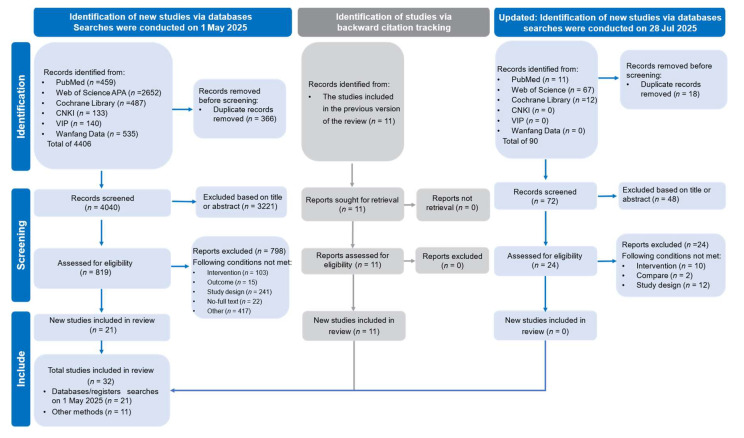
PRISMA flow diagram of study selection process.

A literature search was conducted in six databases: PubMed, Web of Science APA, the Cochrane Library, and three Chinese databases: CNKI (China National Knowledge Infrastructure), VIP (Chinese Scientific Journals Database), and Wanfang Data. The initial search, conducted on 1 May 2025, identified 4406 records. After removing 366 duplicates, 4040 records remained for the title and abstract screening. An additional 11 studies were identified through backward citation tracking of a previous version of the review. An updated search was conducted on 28 July 2025, which yielded 90 records; following the removal of 18 duplicates, 72 records were screened. After a full-text assessment, a total of 32 studies met the inclusion criteria and were included in the meta-analysis [27,28,29,30,31,32,33,34,35,36,37,38,39,40,41,42,43,44,45,46,47,48,49,50,51,52,53,54,55,56,57,58]. These consisted of 21 new studies identified from the May search and 11 studies included from the previous review. No additional studies were included from the July update. The full selection process is shown in Figure 1.

### 3.2. Characteristics of the Included Studies

Among the 32 included studies, 28 were randomized controlled trials (RCTs). The total number of participants across all the studies was 1126, ranging in age from 11–68 years. Among the participants, 786 were male (69.81%), 260 were female (23.09%), and 80 (7.10%) did not explicitly report their sex. Across all the studies, 599 participants received recreational football interventions, whereas 527 were assigned to control conditions. The duration of the intervention programs varied from 4 to 48 weeks. Within individual sessions, the duration of football play ranged from 4 to 30 min, with 2- to 3-min recovery periods between bouts. The total number of sets per session ranged from 1 to 6. Most of the included studies provided detailed information on participant characteristics, study design, intervention protocols, and follow-up arrangements. A summary of the baseline characteristics of the participants in each study is presented in Table 1.

### 3.3. Effects of Recreational Football on Body Composition

#### 3.3.1. Body Weight and Body Mass Index

**Figure 2:**
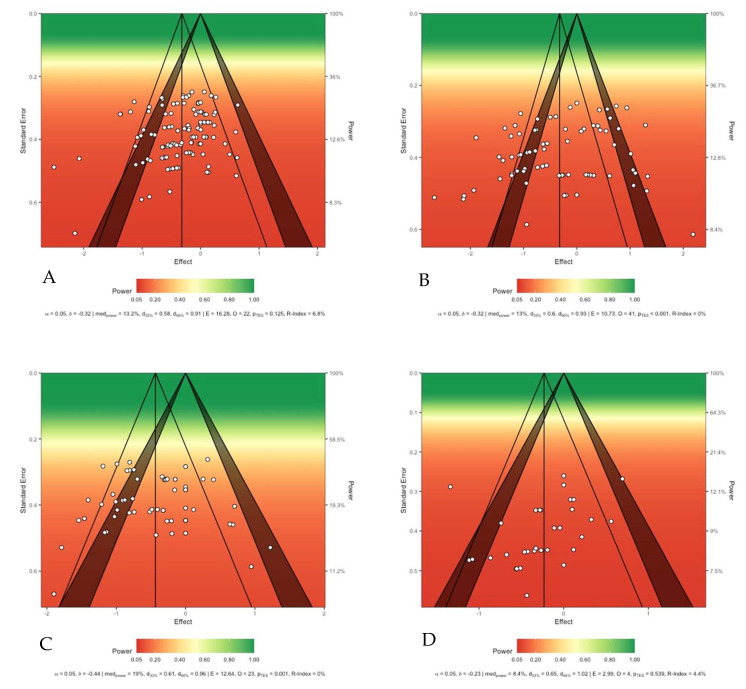
Sunset statistical power charts: (**A**) shows the plot for body composition outcomes; (**B**) shows the plot for resting blood pressure and heart rate; (**C**) shows the plot for lipid metabolism; and (**D**) shows the plot for blood glucose.

**Figure 3:**
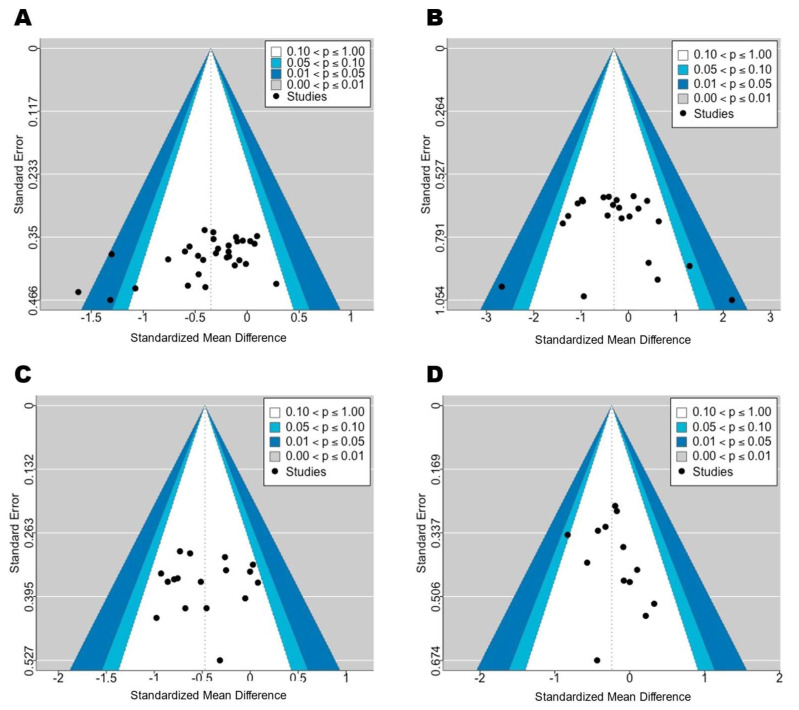
Funnel plots: (**A**) is the funnel plot for body composition outcomes; (**B**) is the funnel plot for resting blood pressure and heart rate; (**C**) is the funnel plot for lipid metabolism; and (**D**) is the funnel plot for blood glucose.

Compared with the control conditions, recreational football training resulted in significant reductions in participants’ body weight (SMD = −0.51 [−0.79 to −0.23]; *p* < 0.01; I^2^ = 67%) and BMI (SMD = −0.41 [−0.66 to −0.15]; *p* < 0.01; I^2^ = 59%). For detailed forest plots, please refer to Appendix B. Subgroup analyses revealed no statistically significant moderating effects of training-related variables, such as sex, age, weight status, intervention duration, training frequency, or rest interval, on changes in participants’ body weight or BMI (Pb > 0.05). Please see Figure 2 and Figure 3 for the corresponding statistical power diagrams and publication bias risk funnel plots for the combined results of each outcome.

#### 3.3.2. Body Fat, Fat Mass, and Lean Body Mass

Recreational football training significantly reduced the body fat percentage (SMD = −0.53 [−0.72 to −0.35]; *p* < 0.01; I^2^ = 32%) and total fat mass (SMD = −0.40 [−0.60 to −0.20]; *p* < 0.01; I^2^ = 20%) of the participants, whereas the lean body mass increased significantly (SMD = 0.18 [0.03 to 0.32]; *p* = 0.02; I^2^ = 0%). For detailed forest plots in this regard, please refer to Appendix B.

Subgroup analyses indicated that age significantly moderated the effect of recreational football on the participants’ body fat percentage (Pb = 0.04). The most substantial improvements were observed in participants aged ≤18 years (g = −0.90 [−1.28 to −0.51]), followed by those aged 18–45 years (g = −0.66 [−1.03 to −0.30]), whereas the effect on individuals aged ≥45 years was relatively small (g = −0.38 [−0.59 to −0.16]).

In regard to fat mass, only sex emerged as a significant moderator (Pb = 0.03). Females presented the greatest reduction (g = −1.16 [−1.74 to −0.58]), followed by males (g = −0.29 [−0.53 to −0.05]); the mixed-sex subgroup presented a marginally significant effect (*p* = 0.08). No significant subgroup differences were observed for lean body mass.

#### 3.3.3. Waist Circumference

Waist circumference was significantly lower in the recreational football group than in the control group (SMD = −1.43 [−2.24 to −0.61]; *p* < 0.01; I^2^ = 86%). For detailed forest plots in this regard, please refer to Appendix B.

Subgroup analyses identified sex (Pb < 0.01) and age (Pb < 0.01) as significant moderators. Among the sex-based subgroups, females presented the greatest reduction (g = −3.06 [−4.16 to −1.96]), followed by males (g = −2.05 [−3.57 to −0.54]), whereas the mixed-sex subgroup presented smaller effects (g = −0.73 [−1.31 to −0.15]).

**Figure 4:**
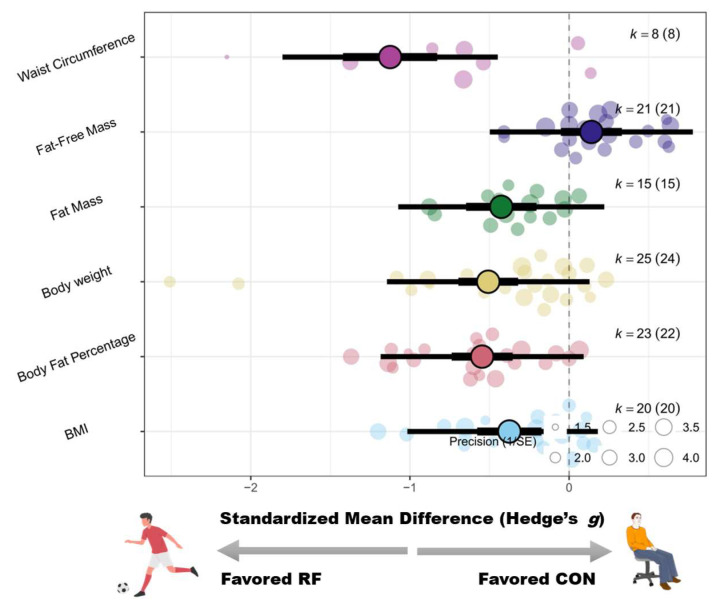
Orchard plot of the effects of recreational football on body composition.

The age-based analysis indicated significant reductions in both the ≤18 years group (g = −1.75 [−3.04 to −0.46]) and the 18–45 years group (g = −2.09 [−3.39 to −0.79]), but not in the ≥45 years group (g = −0.24 [−0.78 to 0.29]). A schematic diagram of the orchards summarizing each outcome indicator is shown in Figure 4.

### 3.4. Effects of Recreational Football on Cardiometabolic Health

#### 3.4.1. Resting Blood Pressure and Heart Rate

Recreational football training was associated with significant improvements in resting cardiovascular parameters. Systolic blood pressure was reduced in the participants (SMD = −0.59 [−0.99 to −0.18]; *p* < 0.01; I^2^ = 82%), as were their diastolic blood pressure (SMD = −0.75 [−1.10 to −0.39]; *p* < 0.01; I^2^ = 78%) and mean arterial pressure (SMD = −0.91 [−1.37 to −0.46]; *p* < 0.01; I^2^ = 70%). The resting heart rate of the participants was also significantly decreased (SMD = −0.85 [−1.42 to −0.27]; *p* < 0.01; I^2^ = 83%). For detailed forest plots in this regard, please refer to Appendix C.

Subgroup analyses revealed that sex significantly moderated the effects on participants’ systolic and diastolic blood pressure (Pb < 0.01 for both). Greater reductions were observed in females for both systolic (g = −1.17 [−1.68 to −0.66]) and diastolic pressure (g = −0.88 [−1.37 to −0.38]), followed by males (systolic: g = −0.52 [−0.96 to −0.09]; diastolic: g = −0.98 [−1.39 to −0.57]). No significant effects were found in the mixed-sex subgroup.

In regard to resting heart rate, sex (Pb = 0.02), weight status (Pb = 0.04), and rest interval (Pb = 0.02) significantly moderated the intervention effects. Compared with females, male participants presented greater reductions (g = −1.04 [−1.86 to −0.21]) (g = −0.78 [−1.27 to −0.30]). Greater effects were observed in the obese subgroup (g = −1.83) than in the overweight subgroup (g = −0.54), with only the obese subgroup reaching statistical significance (*p* < 0.01). Interventions with ≤2 min rest intervals had significant effects (g = −1.30 [−2.33–0.28]), whereas interventions with >2 min rest intervals did not (g = 0.19 [-0.43–0.81]).

#### 3.4.2. Peak/Maximal Oxygen Uptake

**Figure 5:**
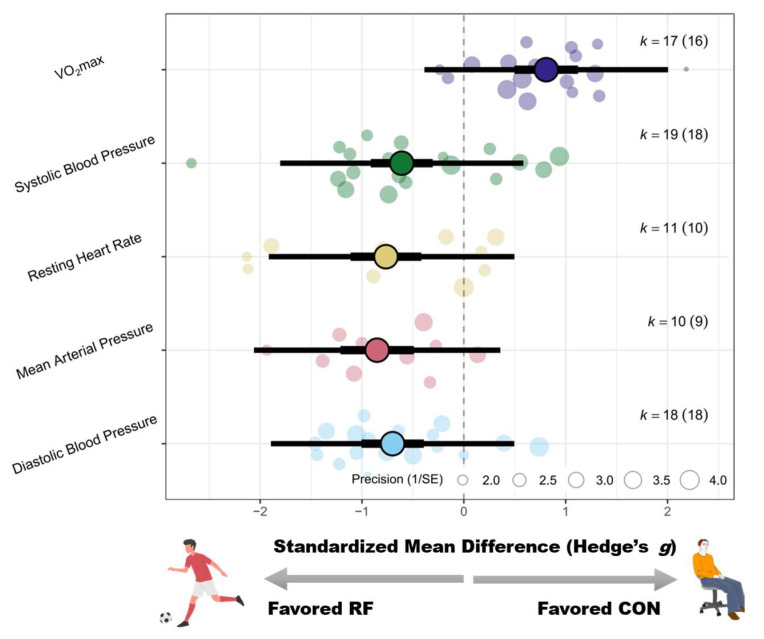
Orchard plot of the effects of recreational football on blood pressure and VO_2_max.

Recreational football significantly increased the peak or maximal oxygen uptake of the participants (SMD = 0.81 [0.54–1.09]; *p* < 0.01; I^2^ = 56%). For detailed forest plots in this regard, please refer to Appendix C. Subgroup analyses revealed that only sex significantly moderated this effect (Pb = 0.03), with mixed-sex groups showing the greatest improvement (g = 1.31 [0.66 to 1.96]), followed by males (g = 0.70 [0.43 to 0.98]). A schematic diagram of the orchards summarizing each outcome indicator is shown in Figure 5.

#### 3.4.3. Lipid Metabolism

Significant improvements were observed in regard to several lipid parameters following recreational football training. Total cholesterol (SMD = −0.62 [−0.91 to −0.34]; *p* < 0.01; I^2^ = 57%), LDL cholesterol (SMD = −0.58 [−0.88 to −0.29]; *p* < 0.01; I^2^ = 58%), and triglycerides (SMD = −0.61 [−0.87 to −0.36]; *p* < 0.01; I^2^ = 41%) were significantly reduced in participants. However, the effect on HDL cholesterol was not statistically significant (SMD = 0.24 [−0.10 to 0.57]; *p* = 0.17; I^2^ = 64%). For detailed forest plots in this regard, please refer to Appendix D.

Subgroup analyses indicated that sex, age, and training frequency significantly moderated total cholesterol outcomes. Greater reductions were found in mixed-sex samples (g = −1.38 [−1.91 to −0.85]), followed by females (g = −0.63 [−1.28 to 0.02]) and males (g = −0.34 [−0.64 to −0.04]). With respect to age, participants ≤18 years showed the greatest improvement (g = −1.25 [−1.98 to −0.52]), followed by those ≥45 years (g = −0.79 [−1.18 to −0.40]); the 18–45 years group showed no significant change (g = −0.14 [−0.45 to 0.18]). A weekly frequency of >2 sessions was more effective (g = −1.01 [−1.48 to −0.55]) than ≤2 sessions (g = −0.33 [−0.58 to −0.08]).

In regard to LDL cholesterol, sex (Pb = 0.02), weight status (Pb = 0.04), and age (Pb < 0.01) significantly moderated the intervention effects. The mixed-sex group had the greatest improvement (g = −1.10 [−1.47 to −0.72]), followed by the male group (g = −0.36 [−0.73 to 0.02]; *p* = 0.06). Compared with overweight individuals, obese participants had greater effects (g = −0.89 vs. −0.32), with significance reached only in the obese group (*p* < 0.01). According to age, significant effects were found in the ≤18 years group (g = −0.89 [−1.36 to −0.42]) and the ≥45 years group (g = −0.85 [−1.17 to −0.54]), but not in the 18–45 years group (g = −0.05; *p* = 0.80).

In regard to triglycerides, sex (Pb = 0.04), age (Pb < 0.01), and training frequency (Pb < 0.01) significantly moderated the effects. The mixed-sex group showed the greatest improvement (g = −1.03 [−1.38 to −0.67]), followed by females (g = −0.78 [−1.27 to −0.30]) and males (g = −0.30 [−0.72 to 0.11]). The ≤18 years group showed the greatest benefit (g = −1.25 [−1.71 to −0.80]), followed by the ≥45 years group (g = −0.65 [−0.92 to −0.37]), with no significant change in the 18–45 years group (g = −0.12 [−0.51 to 0.27]). Interventions conducted >2 times/week were more effective (g = −1.03) than those conducted ≤2 times/week (g = −0.28).

**Figure 6:**
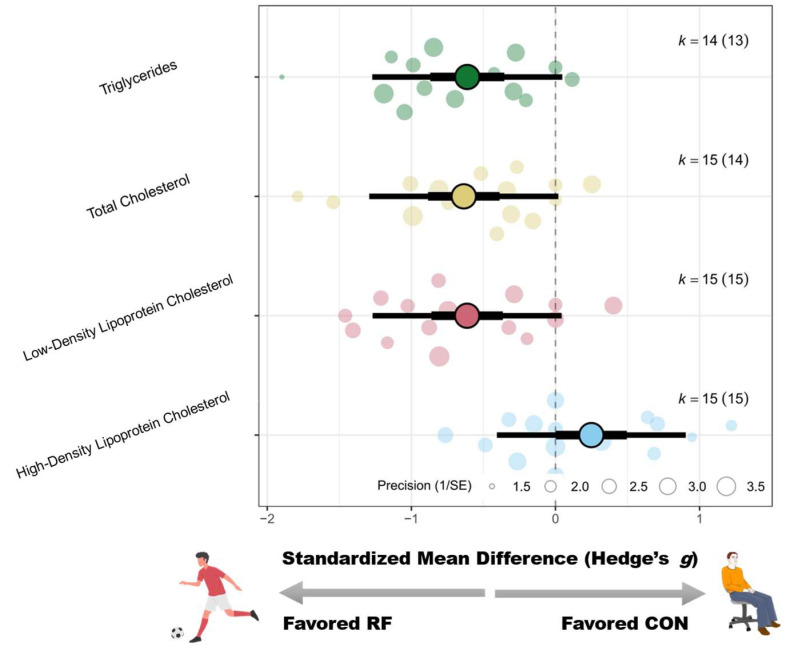
Orchard plot of the effects of recreational football on lipid metabolism.

In regard to HDL cholesterol, sex (Pb < 0.01) and training frequency (Pb = 0.02) moderated the intervention effect. The female participants showed the most significant improvement (g = 2.18 [1.25–3.11]), followed by the mixed-sex participants (g = 0.31 [−0.05–0.67]), whereas no change was observed in the male participants (g = −0.00 [−0.35–0.34]). Sessions held >2 times/week led to significant improvements (g = 0.62 [0.11 to 1.12]; *p* = 0.02) compared with those held ≤2 times/week (g = −0.08 [−0.40 to 0.25]). A schematic diagram of the orchards summarizing each outcome indicator is shown in Figure 6.

#### 3.4.4. Glucose Metabolism

Recreational football significantly reduced participants’ fasting insulin levels (SMD = −0.47 [−0.83– −0.11]; *p* = 0.01; I^2^ = 46%). No significant effects were observed for fasting glucose (SMD = −0.21 [−0.57 to 0.14]; *p* = 0.24; I^2^ = 72%), HbA1c (SMD = −0.15 [−0.45 to 0.15]; *p* = 0.33; I^2^ = 0%), or HOMA-IR (SMD = −0.89 [−1.86 to 0.07]; *p* = 0.07; I^2^ = 79%). For detailed forest plots in this regard, please refer to Appendix D.

**Figure 7:**
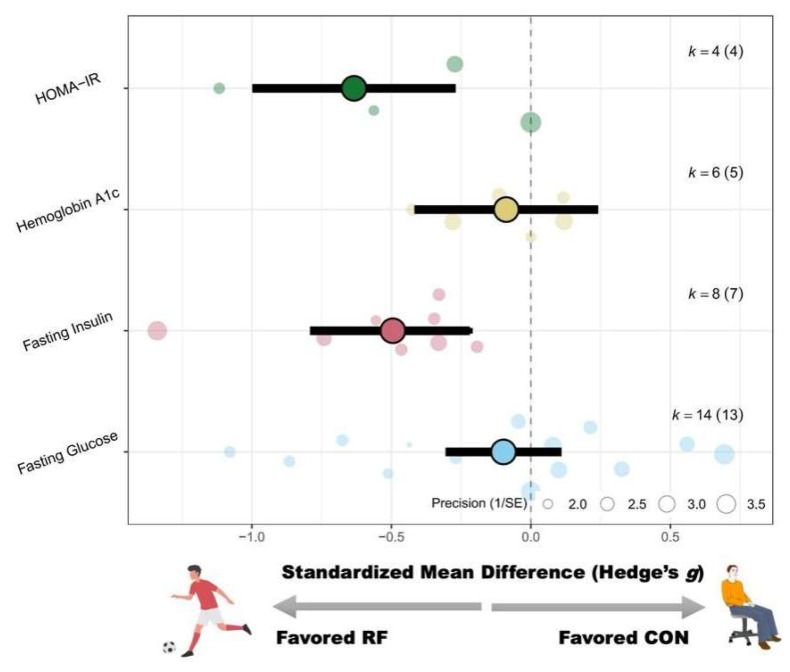
Orchard plot of the effects of recreational football on blood sugar.

Subgroup analyses indicated that intervention duration significantly moderated the effect on fasting insulin (Pb = 0.03). Greater effects were observed in studies with durations >12 weeks (g = −0.83 [−1.31 to −0.36]; *p* < 0.01), whereas no significant effects were found in interventions ≤12 weeks (g = −0.17 [−0.53 to 0.19]; *p* = 0.35). No significant moderators were observed for fasting glucose, HbA1c, or HOMA-IR. A schematic diagram of the orchards summarizing each outcome indicator is shown in Figure 7.

### 3.5. Sensitivity Analysis

To evaluate the robustness of the pooled effect estimates, sensitivity analyses were performed using a leave-one-out approach, in which each included study was sequentially removed to assess its influence on the overall effect size and statistical significance. For the body composition outcomes (e.g., body weight, BMI, body fat percentage), cardiovascular health outcomes (e.g., resting heart rate, systolic/diastolic blood pressure, VO*_2_*max), and glucose–lipid metabolism outcomes (e.g., fasting glucose, insulin, triglycerides, HDL-C, LDL-C), the leave-one-out results consistently demonstrated that the pooled estimates were highly robust. Excluding any single study did not alter the direction, magnitude, or statistical significance of the overall effects. For detailed forest plots and sensitivity statistics for each outcome category, please refer to Appendix E (body composition), Appendix F (cardiovascular health), and Appendix G (glucose–lipid metabolism).

### 3.6. Risk of Bias and Methodological Quality

**Figure 8:**
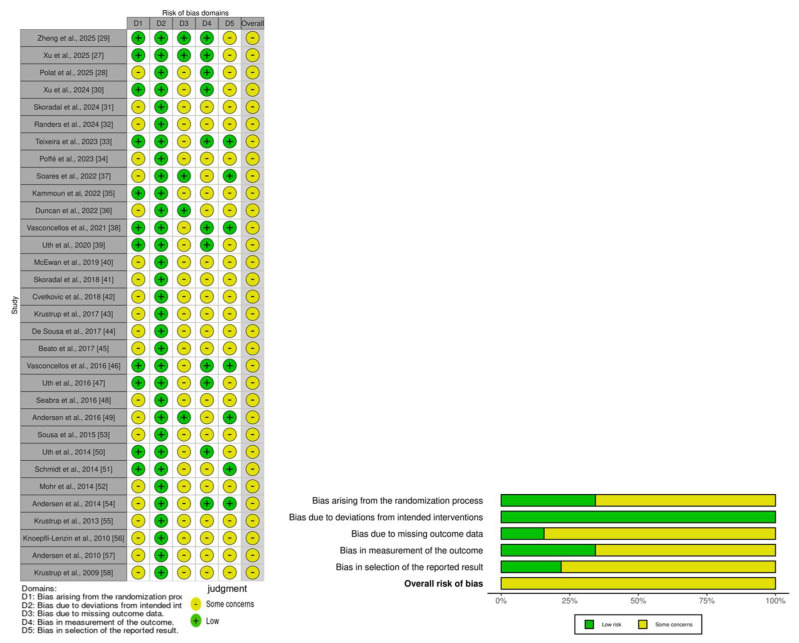
Risk-of-bias assessment diagram [27,28,29,30,31,32,33,34,35,36,37,38,39,40,41,42,43,44,45,46,47,48,49,50,51,52,53,54,55,56,57,58].

The risk of bias was assessed and reported for each included study (Figure 8 and Table 2). Overall, all the studies were judged to present “some concerns” regarding the risk of bias. Specifically, 66% of the studies did not report allocation concealment, thereby presenting some concerns in terms of the randomization process used. Additionally, 84% of the studies had some degree of participant attrition, leading to concerns related to incomplete outcome data. Furthermore, the majority of studies (66%) did not report adequate blinding of the outcome assessment, which also contributed to a judgment of some concerns in this domain.

The methodological quality of the included studies was evaluated via the Physiotherapy Evidence Database (PEDro) scale, as shown in Table 3. The mean PEDro score across all the studies was 5.75, indicating moderate-to-high overall methodological quality. Specifically, 53% of the studies were rated as high quality (score ≥ 6), 44% as moderate quality (scores between 4 and 5), and only 3% were considered low quality (score < 3).

### 3.7. Results of the Certainty of the Evidence

Overall, the certainty of the evidence ranged from very low to moderate across the assessed outcomes, as shown in Table 4. Moderate certainty was found for the effects on the participants’ BMI, waist circumference, diastolic blood pressure, mean arterial pressure, resting heart rate, and VO*_2_*max. In contrast, most other outcomes, including body weight, body fat measures, blood pressure, lipid profiles, glucose metabolism, and insulin resistance, were supported by evidence of low to very low certainty.

## 4. Discussion

This meta-analysis evaluated the effects of recreational football training on body composition and cardiometabolic health outcomes. By synthesizing data from 32 studies, involving 1126 participants, the findings indicate that recreational football is an effective intervention for improving key health markers. Specifically, it resulted in significant reductions in body weight, BMI, body fat percentage, fat mass, waist circumference, blood pressure, resting heart rate, total cholesterol, LDL cholesterol, triglycerides, and fasting insulin, along with modest increases in lean body mass and maximal oxygen uptake. These benefits were most pronounced in females, younger individuals, and those engaging in higher-frequency training. Given its accessible, enjoyable, and group-based format, recreational football shows strong potential as a scalable, community-based strategy for preventing obesity and reducing cardiometabolic risk, in both public health and clinical settings.

### 4.1. Body Composition

The findings revealed that recreational football led to significant reductions in both body weight (SMD = −0.51) and body mass index (BMI; SMD = −0.41) among overweight or obese individuals. These effect sizes exceed those reported by Milanović et al. (2019) [11], who reported only small improvements. This discrepancy may be due to differences in sample composition, as the earlier study included a substantial proportion of participants with a normal weight, whereas the present meta-analysis focused specifically on overweight and obese populations, for whom physiological adaptations may be more pronounced. While these results represent moderate effect sizes, even a 5–10% reduction in body weight has been shown to significantly lower the risk of type 2 diabetes [59,60,61], hypertension [62,63,64], and nonalcoholic fatty liver disease [65], underscoring the clinical relevance of these findings. Such reductions are also widely recommended as initial targets in clinical obesity guidelines [66]. Moreover, most of the included interventions lasted 12 weeks or longer, a duration generally regarded as the minimum threshold for eliciting sustainable improvements in metabolic health. Thus, both the magnitude and timing of the observed weight-related changes support their clinical relevance.

In addition to reducing body weight and BMI, recreational football significantly improved more specific indicators of body composition, including body fat percentage (SMD = −0.53), total fat mass (SMD = −0.40), and lean body mass (SMD = 0.18). These findings align with those of Milanović et al. (2019) [11], who reported substantial reductions in fat mass (−1.72 kg) and modest increases in lean mass. From an epidemiological perspective, even a reduction of 1–2 kg in fat mass has been associated with improved insulin sensitivity, lower systemic inflammation, and favorable lipid profiles [67,68,69]. Lean body mass, particularly skeletal muscle mass, is a key protective factor for metabolic health, as it enhances insulin sensitivity and lipid-glucose metabolism and has been linked to reduced cardiovascular risk [67,68,69]. These improvements, especially in fat mass and lean mass, are not only statistically significant, but are also considered clinically meaningful due to their role in cardiometabolic protection and improved physical function. Subgroup analyses revealed that age significantly moderated the effects of recreational football on body fat percentage, with adolescents (≤18 years) exhibiting the greatest improvements. This may reflect greater metabolic plasticity and heightened anabolic responses during certain developmental stages. In regard to fat mass, sex was identified as a significant moderator, with females demonstrating the most pronounced reductions (g = −1.16), potentially due to higher baseline fat percentages and sex hormone profiles. In contrast, no significant subgroup differences were observed for lean body mass, suggesting relatively uniform benefits across demographic groups.

This study is also the first to systematically evaluate the effects of recreational football on waist circumference, revealing a significant reduction in the participants (SMD = −1.43). Given that even a 2–3 cm decrease in waist circumference has been linked to reductions in visceral adipose tissue and cardiometabolic risk, including improved insulin sensitivity and reduced hepatic fat content, the observed changes are considered clinically meaningful [70,71,72]. Further subgroup analyses revealed that both sex and age significantly moderated the intervention effects. The greatest reductions in waist circumference were observed in women and adults aged 18–45 years, suggesting that these populations may experience greater abdominal fat reductions from recreational football training. In contrast, no significant effect was observed in individuals aged ≥45 years, potentially reflecting age-related shifts in fat distribution, sarcopenia, and reduced metabolic plasticity. Importantly, most interventions lasted 12 weeks or longer, a duration supported by prior evidence as sufficient to elicit changes in visceral fat mass, which closely correlates with waist circumference.

In summary, recreational football has significant benefits for body composition in individuals who are overweight or obese, including reductions in body weight, BMI, body fat, fat mass, and waist circumference, as well as increases in lean mass. However, the magnitude of these benefits may vary by sex and age, underscoring the importance of considering individual characteristics when designing tailored exercise interventions.

### 4.2. Cardiovascular Health

Recreational football training was found to significantly improve cardiovascular parameters, including average reductions of 2.93 mmHg in systolic blood pressure (SBP), 3.21 mmHg in diastolic blood pressure (DBP), and 4.31 beats per minute in terms of the resting heart rate, among overweight or obese individuals. These outcomes correspond to moderate-to-large effect sizes, supporting the feasibility of recreational football as an effective intervention for cardiovascular health. Notably, a reduction of just 2 mmHg in SBP is associated with an approximately 10% lower risk of stroke and a 7% reduction in coronary heart disease risk [73], highlighting the real-world significance of these findings. Similarly, a decrease of 3–4 bpm in the resting heart rate has been linked to lower cardiovascular event risk and all-cause mortality, reinforcing the clinical value of such improvements [74]. While Milanović et al. (2019) [11] reported small-to-moderate improvements in cardiovascular markers in general populations, the present analysis, which focused specifically on individuals at elevated cardiometabolic risk, revealed more pronounced effects, underscoring the intervention’s applicability and value in this population.

Subgroup analyses identified sex as a significant moderator of blood pressure responses, with females demonstrating the greatest reductions in both systolic blood pressure (SBP; g = −1.17) and diastolic blood pressure (DBP; g = −0.88). This may be attributed to greater vascular responsiveness and hormonal modulation during exercise. In regard to the resting heart rate, greater improvements were observed in males (g = −1.04) and obese individuals (g = −1.83), suggesting that these subgroups may have higher baseline autonomic activity and may, thus, experience stronger physiological adaptations to exercise. Additionally, intervention intensity, as indicated by the rest interval duration, was a significant factor; participants with rest intervals of ≤2 minutes exhibited substantial reductions in their resting heart rate (g = −1.30), whereas those with longer intervals (>2 minutes) showed no significant changes. These findings suggest that short-rest, high-density formats may be more effective in eliciting clinically meaningful improvements in cardiovascular function.

This meta-analysis also demonstrated that recreational football led to significant improvements in the peak or maximal oxygen uptake (VO_2_max), with a standardized mean difference of 0.81, indicating a moderate-to-large effect. This finding is consistent with the results by Milanović et al. (2015) [12], who reported a mean increase of 3.51 mL/kg/min in participants’ VO_2_max after 12 weeks of football training, with benefits observed across different age groups, sexes, and health statuses. These increases are clinically meaningful, as a 1 Metabolic Equivalent improvement in the VO_2_max (approximately 3.5 mL/kg/min) is linked to a 19% reduction in cardiovascular mortality [75]. Notably, Milanović et al. [12] also highlighted substantial benefits for both men (ES = 1.22) and women (ES = 0.96), with older adults achieving VO_2_max gains as high as 15–18%. In the present meta-analysis, the largest effect size was observed in mixed-sex groups (g = 1.31), suggesting that the social interaction and motivational components inherent in group-based recreational football may enhance adherence and training responsiveness, particularly in heterogeneous populations. Moreover, the VO_2_peak, as a direct measure of cardiorespiratory fitness, should be regarded as a clinical vital sign [76], since a low VO_2_peak is strongly associated with an increased risk of metabolic diseases [77], cardiovascular disease [78], and certain cancers.

From a physiological perspective, recreational football involves inherently high-intensity and intermittent activity, with heart rates frequently exceeding 90% HR_max_. This intensity level surpasses that of traditional aerobic exercises, such as jogging. The repeated exposure to such high physiological loads promotes meaningful adaptations in cardiac output, oxygen delivery, and muscle-level oxygen utilization. In addition, recreational football offers unique motivational advantages. Compared with conventional training methods, it tends to be more enjoyable and socially engaging through team-based play, which may foster greater adherence and long-term commitment to physical activity. These factors are critical for sustaining the benefits of any intervention over time.

In conclusion, recreational football training delivers substantial cardiovascular improvements in overweight or obese individuals, including reductions in blood pressure and resting heart rate, as well as increases in VO_2_max. Observed variations in outcomes based on sex, weight status, and specific training characteristics, such as the rest interval duration, further emphasize the importance of tailoring exercise programs to individual needs to optimize the results.

### 4.3. Lipid and Glucose Metabolism

Recreational football training was associated with significant improvements in several indicators of lipid metabolism, demonstrating moderate effect sizes in reducing total cholesterol (SMD = −0.62), low-density lipoprotein (LDL) cholesterol (SMD = −0.58), and triglycerides (TG) (SMD = −0.61). These improvements are clinically meaningful, given that elevated LDL and TG levels are well-established independent risk factors for cardiovascular disease. Even modest reductions in these markers are associated with a reduced incidence of atherosclerosis and related cardiovascular events. However, the increase in high-density lipoprotein (HDL) cholesterol was not statistically significant (SMD = 0.24; *p* = 0.17). These findings are partially aligned with previous research. Although Milanović et al. (2019) [11] did not systematically assess lipid profiles, they highlighted that recreational football resembles high-intensity interval training (HIIT) because of its structure, which includes repeated high-intensity efforts and frequent sprints. Prior studies on HIIT have reported substantial improvements in lipid profiles, particularly in TG and LDL levels [79,80,81]. Mechanistically, the repeated muscular contractions and elevated energy demands inherent to football training may stimulate lipoprotein lipase activity and enhance lipid oxidation capacity, providing a physiological rationale for the observed improvements in lipid metabolism [82].

Subgroup analyses identified several key moderators influencing lipid-related outcomes. Sex emerged as a consistent moderator, with mixed-sex groups showing the greatest reductions in total cholesterol (g = −1.38), LDL cholesterol (g = −1.10), and triglycerides (g = −1.03), possibly due to enhanced social interaction and motivational dynamics in heterogeneous training environments. In contrast, females presented the most pronounced increases in HDL cholesterol (g = 2.18), far exceeding those observed in males or mixed-sex groups, suggesting sex-specific responsiveness in anti-atherogenic lipid profiles. Age also played a substantial moderating role; adolescents (≤18 years) demonstrated the greatest improvements in total cholesterol, LDL, and triglycerides, potentially reflecting greater metabolic plasticity and training responsiveness. In comparison, participants aged 18–45 years presented limited changes to these markers. Intervention frequency was another critical factor. Compared with those who trained two or fewer times per week, those who trained more than two times per week experienced significantly greater reductions in total cholesterol (g = −1.01) and triglycerides (g = −1.03), as well as larger increases in HDL cholesterol (g = 0.62). These findings suggest that improvements in lipid metabolism as a result of recreational football are dose dependent and may require reaching a certain threshold of training volume to trigger adaptive mechanisms, such as lipid mobilization, transport, and lipoprotein remodeling. These adaptations likely involve pathways including AMP-activated protein kinase activation, increased lipoprotein lipase activity, and increased mitochondrial oxidative capacity, processes that typically necessitate sustained and sufficient exercise stimuli [83,84,85].

In terms of glucose metabolism, recreational football was associated with a significant reduction in fasting insulin levels (SMD = −0.47), indicating improved insulin sensitivity. Clinically, reductions in fasting insulin reflect enhanced peripheral glucose uptake and reduced insulin resistance, key protective factors against the progression of type 2 diabetes and metabolic syndrome. Subgroup analyses revealed that intervention duration significantly moderated this effect (Pb = 0.03). Specifically, interventions lasting more than 12 weeks produced a marked reduction in fasting insulin (g = −0.83; *p* < 0.01), whereas shorter interventions (≤12 weeks) did not yield significant changes (g = −0.17; *p* = 0.35). These results suggest that longer-term engagement in recreational football is likely required to induce meaningful metabolic adaptations, such as the increased expression of glucose transporter proteins in skeletal muscle, enhanced oxidative enzyme activity, and improved cellular insulin signaling pathways [86,87].

However, recreational football did not significantly improve other markers of glucose metabolism, such as fasting glucose, glycated hemoglobin (HbA1c), or homeostatic model assessment of insulin resistance (HOMA-IR), and no subgroup-level moderators were identified for these outcomes. One plausible explanation is that many participants, despite being overweight or obese, may have had baseline glucose levels within the normal range, thus limiting the potential for observable improvements. Additionally, glycated hemoglobin reflects average blood glucose over 8 to 12 weeks, and changes in this marker may require interventions of higher intensity or greater duration or targeting individuals with pre-existing glucose dysregulation to yield measurable effects. It is also important to consider that glucose homeostasis is regulated by multiple systems, including pancreatic hormone secretion, hepatic glucose output, and dietary carbohydrate intake [88,89,90,91,92,93], which may not be sufficiently altered by exercise alone. In contrast, abnormalities in lipid metabolism may have been more common in the study population, potentially explaining the more robust effects observed for lipid-related outcomes. Future studies should consider enrolling participants with impaired glucose regulation or prediabetes and extending the intervention duration to better evaluate the effects of recreational football on glycemic control.

In summary, recreational football training effectively improved lipid metabolism in overweight or obese individuals, particularly through reductions in total cholesterol, triglycerides, and LDL cholesterol. Females exhibited greater improvements in HDL levels, and more frequent training (i.e., >2 sessions per week) emerged as a key determinant of lipid-related benefits. Recreational football also significantly reduced fasting insulin, suggesting enhanced insulin sensitivity, although it did not significantly impact direct measures of glycemic control, such as fasting glucose or HbA1c.

### 4.4. Limitations and Future Directions

Several limitations should be acknowledged when interpreting the findings of this meta-analysis. First, considerable heterogeneity (I^2^ > 70%) was observed across several outcomes, likely stemming from variations in participant characteristics, training frequency, intervention duration, and baseline fitness levels. In addition, methodological inconsistencies, such as a lack of blinding, unclear randomization procedures, and allocation concealment, were noted in several trials, as revealed in our PEDro-based quality assessment (please refer to Table 2). These factors may compromise the internal validity and generalizability of the reported effects. Second, the potential for publication bias remains, and many included trials had small sample sizes, limiting the statistical power and increasing the risk of random error or inflated effect estimates. Third, the short duration of most interventions (4–16 weeks) offers limited insight into the long-term sustainability of the benefits, behavioral adherence, or health maintenance. Fourth, most studies used non-exercise controls, limiting the ability to compare recreational football with other established training modalities, such as resistance or aerobic training. Finally, the existing evidence base is predominantly derived from young to middle-aged males, with limited data on females, older adults, or individuals with chronic conditions. Moreover, although recreational football is known for its high enjoyment, safety considerations, particularly for middle-aged, low-fitness, or at-risk individuals, remain underreported.

Future research should aim to address several key gaps identified in the current body of evidence. First, in order to reduce methodological heterogeneity, future studies should employ high-quality randomized controlled trials, with standardized intervention protocols and consistent reporting procedures. Second, in order to enhance statistical robustness and reduce the risk of random error, larger sample sizes are strongly recommended. Third, long-term follow-up studies are needed to evaluate the durability of the metabolic effects, participant adherence over time, and any delayed benefits or adverse outcomes. Fourth, comparative research is warranted to assess the relative efficacy of recreational football compared with other exercise modalities, such as aerobic or resistance training, thereby informing more precise exercise prescriptions. Fifth, future investigations should aim to include more diverse participant populations, especially women, older adults, and individuals with diagnosed metabolic conditions, to improve the generalizability of the results. Sixth, standardized reporting of adverse events and injury risk should be prioritized to ensure a clearer understanding of the safety profile of recreational football, particularly among lower fitness or middle-aged individuals. Finally, mechanistic studies using biomarkers and physiological molecular techniques could help uncover the underlying pathways through which recreational football exerts its health benefits.

### 4.5. Practical Implications

To effectively implement recreational football interventions for untrained, overweight, and obese individuals, a minimum intervention period of 12 weeks is recommended, comprising three structured sessions per week. Each session should include a warm-up phase, a football-based training component, and a cool down, with a total session duration of approximately 45~60 min. The warm-up should follow the FIFA 11+ protocol [94], lasting ~10 min, and incorporate running-based drills, core activation, and strength and balance exercises to enhance neuromuscular control and reduce injury risk. The core training component should involve four bouts of small-sided football, each lasting 4 to 6 min, interspersed with approximately 2 min of rest, yielding 20 to 30 min of total active play. Exercise intensity should be progressively increased, targeting an average of ~80% HR_max_. For older adults or individuals without prior exercise experience, it is advisable to begin at low–moderate intensities, with extended rest periods, gradually advancing to higher intensities and shorter recovery durations to ensure safe and effective overload. The cool-down phase, lasting 5 to 10 min, should include low-intensity activity and stretching to promote recovery. The number of players per team (e.g., 3v3 to 5v5) and the pitch dimensions should be adjusted according to participants’ fitness levels and safety considerations. Recreational football should remain enjoyable and socially engaging, fostering greater adherence and long-term participation. Although subgroup analyses suggest that younger adults, females, and obese individuals may experience enhanced benefits, the intervention is considered suitable for individuals across age, sex, and body composition categories, and broad participation is strongly encouraged. Such high-intensity intermittent exercise is well proven to benefit cardiometabolic health [95,96], and recreational football provides a potential way to achieve this. Multiple daily bouts may also help counteract the harms of prolonged sitting [97,98,99].

## 5. Conclusions

This meta-analysis demonstrated that recreational football improved the body composition and cardiometabolic health among overweight and obese individuals. The intervention led to reductions in body weight, BMI, body fat, waist circumference, blood pressure, LDL cholesterol, triglycerides, and fasting insulin, alongside increases in lean body mass and maximal oxygen uptake. These effects were generally more pronounced in females, younger participants, and those engaging in more frequent training. Based on the current evidence, recreational football has emerged as a promising lifestyle intervention for supporting weight management and lowering cardiometabolic risk, particularly in overweight and obese populations. Its structured yet adaptable format also highlights its potential for integration into community health programs and clinical practice.

## Figures and Tables

**Table 1:** Characteristics of the participants in the studies included.

First Author	Design	Participants	Height (cm)	Weight (kg)	BMI (kg/m^2^)	SSG Sessions	Fre	Wk	PEDro
Zheng, 2025 [29]	RCT	SSG: 14, Male/Female (%): 100% Con: 15, Male/Female (%): 100% SSG Age: 15.1, Con Age: 15.1	SSG: 165.4 Con: 168.5	SSG: 77.4 Con: 79.5	SSG: 28.3 Con: 28.0	Total Sets: 4 Duration per Set: 4 (min) Intermittent Recovery: 2 (min) Total Exercise Time: 16 (min) Number of Competitors: 3v3	2	6	8
Xu, 2025 [27]	RCT	SSG: 30, Male/Female (%): 50% Con: 20, Male/Female (%): 50% SSG Age: 19.9, Con Age: 19.65	SSG: 169 Con: 168	SSG: 75.1 Con: 75.95	SSG: N/A Con: N/A	Total Sets: 4 Duration per Set: 5 (min) Intermittent Recovery: 2 (min) Total Exercise Time: 20 (min) Number of Competitors: 2v2, 4v4	3	8	8
Polat, 2025 [28]	RCT	SSG: 28, Male/Female (%): 100% Con: 29, Male/Female (%): 100% SSG Age: 65, Con Age: 66	SSG: 170.6 Con: 167.4	SSG: 80.1 Con: 82.7	SSG: 27.3 Con: 27.9	Total Sets: 3 Duration per Set: 15 (min) Intermittent Recovery: 2 (min) Total Exercise Time: 45 (min) Number of Competitors: 4v4–7v7	2	14	7
Xu, 2024 [30]	RCT	SSG: 15, Male/Female (%): 100% Con: 15, Male/Female (%): 100% SSG (female): 15, Male/Female (%): 0% Con (female): 15, Male/Female (%): 0% SSG Age: 20.1, Con Age: 20.3 SSG (female) Age: 20.1 Con (female) Age: 20.3	SSG: 172 Con: 162 SSG (female): 172 Con (female): 162	SSG: 94.4 Con: 78.2 SSG (female): 94.4 Con (female): 78.2	SSG (female): 31.3 Con (female): 29.5	Total Sets: 2 Duration per Set: 15 (min) Intermittent Recovery: 3 (min) Total Exercise Time: 30 (min) Number of Competitors: 5v5	5	16	8
Skoradal, 2024 [31]	RCT	SSG: 38, Male/Female (%): 47.4% Con: 28, Male/Female (%): 50% SSG Age: 65.9, Con Age: 66.9	SSG: 171 Con: 171	SSG: 80.0 Con: 76.8	SSG: 27.5 Con: 26.0	Total Sets: N/A Duration per Set: N/A Intermittent Recovery: N/A Total Exercise Time: 35–40 (min) Number of Competitors: 4v4–7v7	2	12	5
Randers, 2024 [32]	RCT	SSG1&SSG2: 10, Male/Female (%): 100% Con: 10, Male/Female (%): 50% SSG1&SSG2-Age: 30.7, Con-Age: 30.7	SSG1&SSG2: 183.8 Con: 183.8	SSG1&SSG2: 90.9 Con: 90.9	SSG1&SSG2: N/A Con: N/A	Total Sets: 4 Duration per Set: 12 (min) Intermittent Recovery: 3 (min) Total Exercise Time: 48 (min) Number of Competitors: SSG1: 2v2, 3v3 SSG2: 2v2–5v5	2	12	4
Teixeira, 2023 [33]	RCT	SSG: 20, Male/Female (%): 100% Con: 19, Male/Female (%): 100% SSG Age: 48, Con Age: 51	SSG: N/A Con: N/A	SSG: N/A Con: N/A	SSG: 32.2 Con: 32.0	Total Sets: 4 Duration per Set: 10 (min) Intermittent Recovery: 2 (min) Total Exercise Time: 40 (min) Number of Competitors: 3v3–6v6	2	3 Months	8
Poffé, 2023 [34]	RCT	SSG: 15, Male/Female (%): 93.3% Con: 18, Male/Female (%): 94.4% SSG Age: 63.7, Con Age: 63.2	SSG: 174.0 Con: 175.9	SSG: 81.8 Con: 81.4	SSG: 27.0 Con: 26.3	Total Sets: 2 Duration per Set: 15 (min) Intermittent Recovery: 3 (min) Total Exercise Time: 30 (min) Number of Competitors: 4v4, 5v5	2	10	5
Soares, 2022 [37]	RCT	SSG: 13, Male/Female (%): 61% Con: 13, Male/Female (%): 69% SSG Age: 60.5, Con Age: 60.2	SSG: N/A Con: N/A	SSG: 87.8 Con: 85.5	SSG: 33.2 Con: 33.3	Total Sets: 2 Duration per Set: 12 (min) Intermittent Recovery: 3 (min) Total Exercise Time: 24 (min) Number of Competitors: 3v3–7v7	3	12	6
Kammoun, 2022 [35]	RCT	SSG: 18, Male/Female (%): N/A Con: 13, Male/Female (%): N/A SSG Age: 53.70, Con Age: 52.75	SSG: 177.75 Con: 175.46	SSG: 89.1 Con: 84.6	SSG: 28.2 Con: 27.9	Total Sets: 1 Duration per Set: N/A Intermittent Recovery: N/A Total Exercise Time: 20 (min) Number of Competitors: 4v4, 5v5	3	4	7
Duncan, 2022 [36]	CT	SSG: 13, Male/Female (%): 100% Con: 13, Male/Female (%): 100% SSG Age: 66, Con Age: 66	SSG: N/A Con: N/A	SSG: N/A Con: N/A	SSG: 28.8 Con: 26.4	Total Sets: 6 Duration per Set: 4 (min) Intermittent Recovery: 4 (min) Total Exercise Time: 24 (min) Number of Competitors: 3v3, 4v4	2	12	4
Vasconcellos, 2021 [38]	RCT	SSG: 6, Male/Female (%): 33.3% Con: 7, Male/Female (%): 28.6% SSG Age: 13.9, Con Age: 14.7	SSG: 160.9 Con: 163.1	SSG: 85.1 Con: 84.7	SSG: 30.5 Con: 30.8	Total Sets: N/A Duration per Set: N/A Intermittent Recovery: N/A Total Exercise Time: 40 (min) Number of Competitors: 2v2–4v4	3	12	7
Uth, 2020 [39]	RCT	SSG: 33, Male/Female (%): N/A Con: 16, Male/Female (%): N/A SSG Age: 47.4, Con Age: 50.0	SSG: N/A Con: N/A	SSG: 72.6 Con: 76.8	SSG: 25.5 Con: 26.4	Total Sets: 4 Duration per Set: 7 (min) Intermittent Recovery: 2 (min) Total Exercise Time: 28 (min) Number of Competitors: 4v4, 5v5	2	12 Months	6
McEwan, 2019 [40]	CT	SSG: 9, Male/Female (%): 100% Con: 7, Male/Female (%): 100% SSG Age: 56, Con Age: 60	SSG: 100.6 Con: 98.0	SSG: N/A Con: N/A	SSG: 33.4 Con: 32.1	Total Sets: N/A Duration per Set: N/A Intermittent Recovery: N/A Total Exercise Time: N/A Number of Competitors: N/A	1	8	2
Skoradal, 2018 [41]	RCT	SSG: 27, Male/Female (%): 50.9% Con: 23, Male/Female (%): 50.9% SSG Age: 60, Con Age: 62	SSG: 172 Con: 170	SSG: 85.0 Con: 89.9	SSG: 28.6 Con: 30.9	Total Sets: 2 Duration per Set: 30 (min) Intermittent Recovery: 3 (min) Total Exercise Time: 60 (min) Number of Competitors: 4v4–6v6	2	32	5
Cvetkovic, 2018 [42]	RCT	SSG: 10, Male/Female (%): 100% Con: 14, Male/Female (%): 100% SSG Age: 11–13, Con Age: 11–13	SSG: 157.9 Con: 162.7	SSG: 63.7 Con: 67.4	SSG: 25.4 Con: 25.3	Total Sets: 4 Duration per Set: 8 (min) Intermittent Recovery: 2 (min) Total Exercise Time: 32 (min) Number of Competitors: 5v5–7v7	3	12	4
Krustrup, 2017 [43]	RCT	SSG: 19, Male/Female (%): 0% Con: 12, Male/Female (%): 0% SSG Age: 45, Con Age: 45	SSG: 165 Con: 166	SSG: 75.1 Con: 78.9	SSG: N/A Con: N/A	Total Sets: 4 Duration per Set: 12 (min) Intermittent Recovery: N/A Total Exercise Time: 48 (min) Number of Competitors: 4v4–8v8	3	12 Months	4
De Sousa, 2017 [44]	RCT	SSG: 22, Male/Female (%): 43.1% Con: 29, Male/Female (%): 43.1% SSG Age: 61, Con Age: 61	SSG: N/A Con: N/A	SSG: N/A Con: N/A	SSG: 33.0 Con: 32.7	Total Sets: 4 Duration per Set: 4 (min) Intermittent Recovery: 2 (min) Total Exercise Time: 16 (min) Number of Competitors: 3v3–7v7	3	12	4
Beato, 2017 [45]	RCT	SSG: 10, Male/Female (%): 100% Con: 14, Male/Female (%): 100% SSG Age: 42.9, Con Age: 45.6	SSG: 175.1 Con: 174.9	SSG: 82.1 Con: 81.8	SSG: 26.7 Con: 26.7	Total Sets: 1 Duration per Set: N/A Intermittent Recovery: N/A Total Exercise Time: 55 (min) Number of Competitors: 5v5	1	12	5
Vasconcellos, 2016 [46]	RCT	SSG: 10, Male/Female (%): 80% Con: 10, Male/Female (%): 60% SSG Age: 14.1, Con Age: 14.8	SSG: 163.1 Con: 161.2	SSG: 82.2 Con: 86.3	SSG: 30.3 Con: 32.2	Total Sets: 4 Duration per Set: 4 (min) Intermittent Recovery: 2 (min) Total Exercise Time: 16 (min) Number of Competitors: 2v2–4v4	3	12	7
Uth, 2016 [47]	RCT	SSG: 29, Male/Female (%): 100% Con: 28, Male/Female (%): 100% SSG Age: 67.1, Con Age: 66.5	SSG: N/A Con: N/A	SSG: N/A Con: N/A	SSG: 26.6 Con: 27.6	Total Sets: 3 Duration per Set: 15 (min) Intermittent Recovery: N/A Total Exercise Time: 45 (min) Number of Competitors: N/A	3	32	5
Seabra, 2016 [48]	CT	SSG: 29, Male/Female (%): 100% Con: 30, Male/Female (%): 100% SSG Age: 10.5, Con Age: 10.0	SSG: 147.5 Con: 145.3	SSG: 52.5 Con: 53.6	SSG: 23.7 Con: 25.1	Total Sets: N/A Duration per Set: N/A Intermittent Recovery: N/A Total Exercise Time: 40–60 (min) Number of Competitors: N/A	3	6 Months	5
Andersen, 2016 [49]	RCT	SSG: 9, Male/Female (%): 100% Con: 8, Male/Female (%): 100% SSG Age: 68.0, Con Age: 67.4	SSG: 173.3 Con: 179.0	SSG: 77.7 Con: 89.3	SSG: 26.1 Con: 27.9	Total Sets: 4 Duration per Set: 15 (min) Intermittent Recovery: 2 (min) Total Exercise Time: 60 (min) Number of Competitors: 3v3–5v5	2	52	6
Sousa, 2015 [53]	RCT	SSG: 19, Male/Female (%): 52.6% Con: 15, Male/Female (%): 33.3% SSG Age: 48–68, Con Age: 48–68	SSG: N/A Con: N/A	SSG: 88.9 Con: 82.9	SSG: 32.7 Con: 33.1	Total Sets: 2 Duration per Set: 12 (min) Intermittent Recovery: 2 (min) Total Exercise Time: 24 (min) Number of Competitors: 3v3–7v7	3	12	5
Uth, 2014 [50]	RCT	SSG: 21, Male/Female (%): 100% Con: 20, Male/Female (%): 100% SSG Age: 67.1, Con Age: 66.5	SSG: 177.0 Con: 180.8	SSG: 83.4 Con: 89.0	SSG: 26.6 Con: 27.6	Total Sets: 3 Duration per Set: 15 (min) Intermittent Recovery: N/A Total Exercise Time: 45 (min) Number of Competitors: 5v5–7v7	3	12	8
Schmidt, 2014 [51]	RCT	SSG: 9, Male/Female (%): 100% Con: 8, Male/Female (%): 100% SSG Age: 68.0, Con Age: 67.4	SSG: 173.3 Con: 179.0	SSG: 77.7 Con: 89.3	SSG: 26.1 Con: 27.9	Total Sets: 4 Duration per Set: 15 (min) Intermittent Recovery: 2 (min) Total Exercise Time: 60 (min) Number of Competitors: 3v3–5v5	3	12 Months	7
Mohr, 2014 [52]	RCT	SSG: 21, Male/Female (%): 0% Con: 20, Male/Female (%): 0% SSG Age: 45, Con Age: 43	SSG: 165 Con 166	SSG: 79.8 Con: 77.3	SSG: N/A Con: N/A	Total Sets: N/A Duration per Set: N/A Intermittent Recovery: N/A Total Exercise Time: N/A Number of Competitors: 4v4–10v10	3	15	6
Andersen, 2014 [54]	CT	SSG: 12, Male/Female (%): 100% Con: 9, Male/Female (%): 100% SSG Age: 50.6, Con Age: 48.7	SSG: N/A Con: N/A	SSG: N/A Con: N/A	SSG: 30.4 Con: 30.4	Total Sets: 5 Duration per Set: 10 (min) Intermittent Recovery: 2 (min) Total Exercise Time: 50 (min) Number of Competitors: 4v4–6v6	2	24	5
Krustrup, 2013 [55]	RCT	SSG: 22, Male/Female (%): 100% Con: 11, Male/Female (%): 100% SSG Age: 46, Con Age: 46	SSG: N/A Con: N/A	SSG: 97.8 Con: 97.8	SSG: 30.0 Con: 30.0	Total Sets: 4 Duration per Set: 12 (min) Intermittent Recovery: 2 (min) Total Exercise Time: 48 (min) Number of Competitors: 5v5–7v7	2	6 Months	6
Knoepfli-Lenzin, 2010 [56]	RCT	SSG: 15, Male/Female (%): 100% Con: 17, Male/Female (%): 100% SSG Age: 37, Con Age: 38	SSG: N/A Con: N/A	SSG: N/A Con: N/A	SSG: 26 Con: 27	Total Sets: N/A Duration per Set: N/A Intermittent Recovery: N/A Total Exercise Time: 50 (min) Number of Competitors: 3v3–5v5	3	12	6
Andersen, 2010 [57]	RCT	SSG: 15, Male/Female (%): 100% Con: 10, Male/Female (%): 100% SSG Age: 46.7, Con Age: 47.8	SSG: 181 Con: 182	SSG: 100.1 Con: 100.0	SSG: 30.4 Con: 30.0	Total Sets: 2 Duration per Set: 25 (min) Intermittent Recovery: 2 (min) Total Exercise Time: 50 (min) Number of Competitors: 5v5–7v7	2	3 Months	6
Krustrup, 2009 [58]	RCT	SSG: 13, Male/Female (%): 100% Con: 11, Male/Female (%): 100% SSG Age: 20–43, Con Age: 20–43	SSG: N/A Con: N/A	SSG: 84.4 Con: 84.4	SSG: 25.6 Con: 25.6	Total Sets: N/A Duration per Set: N/A Intermittent Recovery: N/A Total Exercise Time: 55 (min) Number of Competitors: 5v5–7v7	2	12	5

**Note:** SSG = small-sided game; Con = control group; Fre = training frequency (sessions per week); Wk = intervention duration (weeks); PEDro = Physiotherapy Evidence Database score (study quality scale); RCT = randomized controlled trial. The height, weight, and BMI values are presented as group means. “Number of Competitors” indicates the format (e.g., 3v3).

**Table 2:** Risk of bias assessment based on the Cochrane RoB 2 tool.

Study	D1	D2	D3	D4	D5	Overall
Zheng et al., 2025 [29]	Low	Low	Low	Low	Some concerns	Some concerns
Xu et al., 2025 [27]	Low	Low	Low	Low	Some concerns	Some concerns
Polat et al., 2025 [28]	Some concerns	Low	Some concerns	Low	Some concerns	Some concerns
Xu et al., 2024 [30]	Low	Low	Some concerns	Low	Some concerns	Some concerns
Skoradal et al., 2024 [31]	Some concerns	Low	Some concerns	Some concerns	Some concerns	Some concerns
Randers et al., 2024 [32]	Some concerns	Low	Some concerns	Some concerns	Some concerns	Some concerns
Teixeira et al., 2023 [33]	Low	Low	Some concerns	Low	Low	Some concerns
Poffé et al., 2023 [34]	Some concerns	Low	Some concerns	Some concerns	Some concerns	Some concerns
Soares et al., 2022 [37]	Some concerns	Low	Low	Some concerns	Low	Some concerns
Kammoun et al., 2022 [35]	Low	Low	Some concerns	Some concerns	Some concerns	Some concerns
Duncan et al., 2022 [36]	Some concerns	Low	Low	Some concerns	Some concerns	Some concerns
Vasconcellos et al., 2021 [38]	Low	Low	Some concerns	Low	Low	Some concerns
Uth et al., 2020 [39]	Low	Low	Some concerns	Low	Some concerns	Some concerns
McEwan et al., 2019 [40]	Some concerns	Low	Some concerns	Some concerns	Some concerns	Some concerns
Skoradal et al., 2018 [41]	Some concerns	Low	Some concerns	Some concerns	Some concerns	Some concerns
Cvetkovic et al., 2018 [42]	Some concerns	Low	Some concerns	Some concerns	Some concerns	Some concerns
Krustrup et al., 2017 [43]	Some concerns	Low	Some concerns	Some concerns	Some concerns	Some concerns
De Sousa et al., 2017 [44]	Some concerns	Low	Some concerns	Some concerns	Some concerns	Some concerns
Beato et al., 2017 [45]	Some concerns	Low	Some concerns	Some concerns	Some concerns	Some concerns
Vasconcellos et al., 2016 [46]	Low	Low	Some concerns	Low	Low	Some concerns
Uth et al., 2016 [47]	Low	Low	Some concerns	Low	Some concerns	Some concerns
Seabra et al., 2016 [48]	Some concerns	Low	Some concerns	Some concerns	Some concerns	Some concerns
Andersen et al., 2016 [49]	Some concerns	Low	Low	Some concerns	Low	Some concerns
Sousa et al., 2015 [53]	Some concerns	Low	Some concerns	Some concerns	Some concerns	Some concerns
Uth et al., 2014 [50]	Low	Low	Some concerns	Low	Some concerns	Some concerns
Schmidt et al., 2014 [51]	Low	Low	Some concerns	Some concerns	Low	Some concerns
Mohr et al., 2014 [52]	Some concerns	Low	Some concerns	Some concerns	Some concerns	Some concerns
Andersen et al., 2014 [54]	Some concerns	Low	Some concerns	Low	Low	Some concerns
Krustrup et al., 2013 [55]	Some concerns	Low	Some concerns	Some concerns	Some concerns	Some concerns
Knoepfli-Lenzin et al., 2010 [56]	Some concerns	Low	Some concerns	Some concerns	Some concerns	Some concerns
Andersen et al., 2010 [57]	Some concerns	Low	Some concerns	Some concerns	Some concerns	Some concerns
Krustrup et al., 2009 [58]	Some concerns	Low	Some concerns	Some concerns	Some concerns	Some concerns

**Note:** The risk of bias for each included study was assessed using the Cochrane Risk of Bias 2.0 (RoB 2) tool for randomized trials. The assessment covered five domains: D1 refers to bias arising from the randomization process, including sequence generation, allocation concealment, and baseline differences; D2 evaluates bias due to deviations from the intended interventions, such as non-adherence, unplanned co-interventions, or a lack of blinding; D3 addresses bias due to missing outcome data, particularly attrition, exclusions, or imbalances in regard to the participants lost to follow-up; D4 considers bias in the measurement of the outcomes, including whether outcome assessors were blinded and whether outcome measures were appropriate and consistent; and D5 reflects bias in the selection of the reported results, such as selective outcome reporting or discrepancies between pre-specified protocols and reported outcomes. The overall risk of bias judgment was derived from the combined appraisal across all five domains and categorized as “Low risk”, “Some concerns”, or “High risk”, following the guidance in the Cochrane Handbook.

**Table 3:** PEDro scale assessment results.

Author, Year	D1	D2	D3	D4	D5	D6	D7	D8	D9	D10	D11	Total
Zheng et al., 2025 [29]	Y	1	1	1	0	0	**1**	1	1	1	1	8
Xu et al., 2025 [27]	Y	1	1	1	0	0	1	1	1	1	1	8
Polat et al., 2025 [28]	Y	1	0	1	0	0	1	1	1	1	1	7
Xu et al., 2024 [30]	Y	1	1	1	0	0	1	1	1	1	1	8
Skoradal et al., 2024 [31]	Y	1	0	0	0	0	0	1	1	1	1	5
Randers et al., 2024 [32]	Y	1	0	0	0	0	0	0	1	1	1	4
Teixeira et al., 2023 [33]	Y	1	1	1	0	0	1	1	1	1	1	8
Poffé et al., 2023 [34]	N	1	0	0	0	0	0	1	1	1	1	5
Soares et al., 2022 [37]	Y	1	0	1	0	0	0	1	1	1	1	6
Kammoun et al., 2022 [35]	Y	1	1	1	0	0	0	1	1	1	1	7
Duncan et al., 2022 [36]	Y	0	0	0	0	0	0	1	1	1	1	4
Vasconcellos et al., 2021 [38]	Y	1	1	1	0	0	1	0	1	1	1	7
Uth et al., 2020 [39]	Y	1	1	0	0	0	1	0	1	1	1	6
McEwan et al., 2019 [40]	Y	0	0	0	0	0	0	0	1	0	1	2
Skoradal et al., 2018 [41]	Y	1	0	0	0	0	0	1	1	1	1	5
Cvetkovic et al., 2018 [42]	Y	1	0	1	0	0	0	0	1	0	1	4
Krustrup et al., 2017 [43]	Y	1	0	0	0	0	0	0	1	1	1	4
De Sousa et al., 2017 [44]	Y	1	0	0	0	0	0	0	1	1	1	4
Beato et al., 2017 [45]	Y	1	0	1	0	0	0	0	1	1	1	5
Vasconcellos et al., 2016 [46]	Y	1	1	1	0	0	1	0	1	1	1	7
Uth et al., 2016 [47]	N	1	0	1	0	0	0	0	1	1	1	5
Seabra et al., 2016 [48]	Y	0	0	1	0	0	0	1	1	1	1	5
Andersen et al., 2016 [49]	Y	1	0	1	0	0	0	1	1	1	1	6
Sousa et al., 2015 [53]	Y	1	0	1	0	0	0	0	1	1	1	5
Uth et al., 2014 [50]	Y	1	1	1	0	0	1	1	1	1	1	8
Schmidt et al., 2014 [51]	Y	1	1	1	0	0	0	1	1	1	1	7
Mohr et al., 2014 [52]	Y	1	0	1	0	0	0	1	1	1	1	6
Andersen et al., 2014 [54]	Y	0	0	0	0	0	1	1	1	1	1	5
Krustrup et al., 2013 [55]	Y	1	0	1	0	0	0	1	1	1	1	6
Knoepfli-Lenzin et al., 2010 [56]	Y	1	0	1	0	0	0	1	1	1	1	6
Andersen et al., 2010 [57]	Y	1	0	1	0	0	0	1	1	1	1	6
Krustrup et al., 2009 [58]	Y	1	0	0	0	0	0	1	1	1	1	5

**Notes:** studies scoring ≥6 are considered high quality, those scoring 4–5 are considered moderate quality, and those scoring ≤3 are considered low quality. 1. Eligibility criteria were specified (not included in the total score). 2. The subjects were randomly allocated to groups (in a crossover study, the subjects were randomly allocated in the order in which the treatments were received). 3. Allocation was concealed. 4. The groups were similar at the baseline in terms of the most important prognostic indicators. 5. All the subjects were blinded. 6. All the therapists who administered the therapy were blinded. 7. All assessors who measured at least one key outcome were blinded. 8. Measures of at least one key outcome were obtained from more than 85% of the subjects initially allocated to the groups. 9. All subjects for whom outcome measures were available received the treatment or control condition as allocated or, where this was not the case, data for at least one key outcome were analyzed according to the “intention to treat”. 10. The results of the between-group statistical comparisons are reported for at least one key outcome. 11. The study provides both point measures and measures of variability for at least one key outcome.

**Table 4:** GRADE assessment results.

Outcome	No. of Participants (Studies)	Certainty Assessment					Standardized Mean Effect (95% CI)	GRADE *
		Risk of Bias	Inconsistency	Indirectness	Imprecision	Other		
Body Composition								
Body Weight	24 (26 RCTs)	Serious	Serious	Not serious	Not serious	None	−0.51 (−0.79 to −0.23)	⨁⨁◯◯ LOW
Body Weight Index	21 (20 RCTs)	Serious	Not serious	Not serious	Not serious	None	−0.41 (−0.66 to −0.15)	⨁⨁⨁◯ MODERATE
Body Fat Percentage	23 (34 RCTs)	Serious	Serious	Not serious	Not serious	None	−0.53 (−0.72 to −0.35)	⨁⨁◯◯ LOW
Fat-Free Mass	21 (20 RCTs)	Serious	Serious	Not serious	Not serious	None	0.18 (0.03 to 0.32)	⨁⨁◯◯ LOW
Fat Mass	16 (16 RCTs)	Serious	Not serious	Not serious	Serious	None	−0.40 (−0.60 to −0.20)	⨁⨁◯◯ LOW
Waist Circumstance	10 (11 RCTs)	Serious	Serious	Not serious	Not serious	Large Effect Size	−1.43 (−2.24 to −0.61)	⨁⨁⨁◯ MODERATE
Cardiovascular Health								
Systolic Blood Pressure	19 (20 RCTs)	Serious	Serious	Not serious	Not serious	None	−0.59 (−0.99 to −0.18)	⨁⨁◯◯ LOW
Diastolic Blood Pressure	19 (19 RCTs)	Serious	Not serious	Not serious	Not serious	None	−0.75 (−1.10 to −0.39)	⨁⨁⨁◯ MODERATE
Mean Arterial Pressure	10 (11 RCTs)	Serious	Not serious	Not serious	Not serious	Large effect size	−0.91 (−1.37 to −0.46)	⨁⨁⨁◯ MODERATE
Resting Heart Rate	11 (12 RCTs)	Serious	Serious	Not serious	Not serious	Large effect size	−0.85 (−1.42 to −0.27)	⨁⨁⨁◯ MODERATE
VO_2_max/VO_2_peak	17 (18 RCTs)	Serious	Serious	Not serious	Not serious	Large effect size	0.81 (0.54 to 1.09)	⨁⨁⨁◯ MODERATE
Glucose and Lipid Metabolism								
Total Cholesterol	15 (16 RCTs)	Serious	Serious	Not serious	Not serious	None	−0.62 (−0.91 to −0.34)	⨁⨁◯◯ LOW
LDL Cholesterol	15 (16 RCTs)	Serious	Serious	Not serious	Not serious	None	−0.58 (−0.88 to −0.29)	⨁⨁◯◯ LOW
Triglyceride	14 (15 RCTs)	Serious	Not serious	Not serious	Not serious	None	−0.61 (−0.87 to −0.36)	⨁⨁◯◯ LOW
Fasting Insulin	8 (9 RCTs)	Serious	Serious	Not serious	Not serious	None	−0.47 (−0.83 to −0.11)	⨁⨁◯◯ LOW
HDL Cholesterol	16 (16 RCTs)	Serious	Serious	Not serious	Serious	None	0.24 (−0.10 to 0.57)	⨁◯◯◯ VERY LOW
Fasting Glucose	14 (15 RCTs)	Serious	Serious	Not serious	Serious	None	−0.21 (−0.57 to 0.14)	⨁◯◯◯ VERY LOW
HbA1c	6 (7 RCTs)	Serious	Serious	Not serious	Serious	None	−0.15 (−0.45 to 0.15)	⨁⨁◯◯ LOW
HOMA-IR	5 (5 RCTs)	Serious	Not serious	Not serious	Serious	Large effect size	−0.89 (−1.86 to 0.07)	⨁⨁◯◯ LOW

* Certainty of evidence according to the Grading of Recommendations, Assessment, Development and Evaluations (GRADE). High: We are very confident in the estimated effect. Moderate: Our confidence in the estimated effect is moderate. Low: We have limited confidence in the estimated effect. Very low: We have very little confidence in the estimated effect.

## Data Availability

The corresponding author of this article will unconditionally provide all the original data supporting the results of this study.

## Appendix A. PRISMA

**Section/Topic**	**#**	**Checklist Item**	**Reported on Page #**
Effects of Recreational Football on Body Composition and Cardiometabolic Health in Overweight or Obese Individuals: A Systematic Review and Meta-Analysis			
Title	1	Identify the report as a systematic review, meta-analysis, or both.	1
Structured summary	2	Provide a structured summary including as applicable: background; objectives; data sources; study eligibility criteria, participants, and interventions; study appraisal and synthesis methods; results; limitations; conclusions and implications of key findings; systematic review registration number.	1
INTRODUCTION			
Rationale	3	Describe the rationale for the review in the context of what is already known.	2
Objectives	4	Provide an explicit statement of questions being addressed with reference to participants, interventions, comparisons, outcomes, and study design (PICOS).	2
METHODS			
Protocol and registration	5	Indicate if a review protocol exists, if and where it can be accessed (e.g., Web address), and, if available, provide registration information, including registration number.	3
Eligibility criteria	6	Specify study characteristics (e.g., PICOS, length of follow-up) and report characteristics (e.g., years considered, language, publication status) used as criteria for eligibility, giving rationale.	4
Information sources	7	Describe all information sources (e.g., databases with dates of coverage, contact with study authors to identify additional studies) in the search and date last searched.	4
Search	8	Present full electronic search strategy for at least one database, including any limits used, such that it could be repeated.	4
Study selection	9	State the process for selecting studies (i.e., screening, eligibility, included in systematic review, and, if applicable, included in the meta-analysis).	5
Data collection process	10	Describe method of data extraction from reports (e.g., piloted forms, independently, in duplicate) and any processes for obtaining and confirming data from investigators.	5
Data items	11	List and define all variables for which data were sought (e.g., PICOS, funding sources) and any assumptions and simplifications made.	6
Risk of bias in individual studies	12	Describe methods used for assessing risk of bias in individual studies (including specification of whether this was conducted at the study or outcome level), and how this information is to be used in any data synthesis.	5
Summary measures	13	State the principal summary measures (e.g., risk ratio, difference in means).	5
Synthesis of results	14	Describe the methods of handling data and combining results of studies, if conducted, including measures of consistency (e.g., I^2^), for each meta-analysis.	6
Section/Topic	#	Checklist Item	Reported on Page #
Risk of bias across studies	15	Specify any assessment of the risk of bias that may affect the cumulative evidence (e.g., publication bias, selective reporting within studies).	5
Additional analyses	16	Describe methods of additional analyses (e.g., sensitivity or subgroup analyses, meta-regression), if conducted, indicating which were prespecified.	6
RESULTS			
Study selection	17	Provide numbers of studies screened, assessed for eligibility, and included in the review, with reasons for exclusions at each stage, ideally using a flow diagram.	7
Study characteristics	18	For each study, present characteristics for which data were extracted (e.g., study size, PICOS, follow-up period) and provide the citations.	7
Risk of bias within studies	19	Present data on the risk of bias in each study and, if available, any outcome level assessment (see item 12).	13
Results of individual studies	20	For all outcomes considered (benefits or harms), present, for each study: (a) simple summary data for each intervention group, (b) effect estimates and confidence intervals, ideally using a forest plot.	6–13
Synthesis of results	21	Present results of each meta-analysis conducted, including confidence intervals and measures of consistency.	6–13
Risk of bias across studies	22	Present results of any assessment of risk of bias across studies (see Item 15).	13
Additional analysis	23	Provide results of additional analyses, if conducted (e.g., sensitivity or subgroup analyses, meta-regression [see Item 16]).	6–13
DISCUSSION			
Summary of evidence	24	Summarize the main findings including the strength of evidence for each main outcome; consider their relevance to key groups (e.g., healthcare providers, users, and policy makers).	16
Limitations	25	Discuss limitations at study and outcome level (e.g., risk of bias), and at review level (e.g., incomplete retrieval of identified research, reporting bias).	23,24
Conclusions	26	Provide a general interpretation of the results in the context of other evidence, and implications for future research.	24
FUNDING			
Funding	27	Describe sources of funding for the systematic review and other support (e.g., supply of data); role of funders for the systematic review.	n/a
